# Stroke Volume Measurements by Echocardiography and Ultrasonic Cardiac Output Monitor in Children: A Prospective Observational Cohort Study

**DOI:** 10.1097/PEC.0000000000003018

**Published:** 2023-07-22

**Authors:** Jiri Fremuth, Michal Huml, Tereza Pomahacova, Jiri Kobr, Stanislav Kormunda, Josef Sykora

**Affiliations:** From the Department of Pediatrics, Faculty Hospital, Faculty of Medicine in Pilsen, Charles University in Prague, Prague, Czech Republic.

**Keywords:** ultrasonic cardiac output monitor, echocardiography, cardiac output

## Abstract

**Objectives:**

Stroke volume (SV) and cardiac output monitoring is a cornerstone of hemodynamic assessment. Noninvasive technologies are increasingly used in children. This study compared SV measurements obtained by transcutaneous Doppler ultrasound techniques (ultrasonic cardiac output monitor [USCOM]), transthoracic echocardiography jugular (TTE-J), and parasternal (TTE-P) views performed by pediatric intensivists (OP-As) with limited training in cardiac sonography (20 previous examinations) and pediatric cardiologists (OP-Bs) with limited training in USCOM (30 previous examinations) in spontaneously ventilating children.

**Methods:**

A single-center study was conducted in 37 children. Each operator obtained 3 sets of USCOM SV measurements within a period of 3 to 5 minutes, followed with TTE measurements from both apical and jugular views. The investigators were blinded to each other's results to prevent visual and auditory bias.

**Results:**

Both USCOM and TTE methods were applicable in 89% of patients. The intraobserver variability of USCOM, TTE-J, and TTE-P were less than 10% in both investigators. The SV measurements by OP-As using USCOM, TTE-J, and TTE-P were 46.15 (25.48) mL, 39.45 (20.65) mL, and 33.42 (16.69) mL, respectively. The SV measurements by OP-Bs using USCOM, TTE-J, and TTE-P were 43.99 (25.24) mL, 38.91 (19.98) mL, and 37.58 (19.81) mL, respectively.

The percentage error in SV with USCOM relative to TTE-J was 36% in OP-As and 37% in OP-Bs. The percentage error in SV with TTE-P was 33% relative to TTE-J in OP-As and 21% in OP-Bs.

**Conclusions:**

Our findings show that the methods are not interchangeable because SV values by USCOM are higher in comparison with the SV values obtained by TTE. Both methods have low level of intraobserver variability. The SV measurements obtained by TTE-P were significantly lower compared with the TTE-J for the operator with limited training in echocardiography. The TTE-P requires longer practice compared with the TTE-J; therefore, we recommend to prefer TTE-J to TTE-P for inexperienced operators.

Hemodynamic monitoring supplies invaluable insights about the patient's hemodynamic status that can help to identify underlying pathophysiological processes and is essential for the correct individual management of critically ill patients.^1^ Cardiac output (CO) measurement is a cornerstone of advanced hemodynamic monitoring; however, physicians are likely insufficiently capable of recognizing a low CO when using clinical examinations. The physician's accuracy in subjectively estimating CO, based on a clinical examination, equals the flip of a coin.^2,3^ Relying on end points (such as a clinical examination, arterial pressure, or urinary output) to assess the response to therapy may potentially lead to inadequate resuscitation or overtreatment.^4,5^ The pulmonary artery catheter (PAC) has long been considered the optimal form of hemodynamic monitoring. In the last decade, noninvasive monitoring technologies have been widely used as an alternative to PAC.

Non-invasive Doppler-based methods have become widely popular and have spread in the intensive care setting. The main interest in echocardiography is that it can be used not only for the measurement of CO but also for the additional assessment of cardiac anatomy and function. However, echocardiography instruments and expertise may not be readily available everywhere and, in most institutions, is still the domain of cardiologists.^6^ With the increasing emphasis on early intervention for critically ill patients, the utility of an easy-to-use, noninvasive hemodynamic monitoring technology became obvious.

A noninvasive method of transcutaneous, continuous-wave Doppler ultrasound measurement of CO was developed, USCOM 1A (Ultrasonic Cardiac Output Monitor, USCOM Ltd, Sydney, Australia). The first step in the hemodynamic management of an acutely ill patient is to determine the adequacy of tissue perfusion and to evaluate the hemodynamic status. Previous studies showed evidence of good interrater reliability, short learning curves, and USCOM feasibility.^7,8^ As classic echocardiography has become more widely available, it is being more commonly used by noncardiologists. In children, there are only 2 previous studies comparing CO by USCOM to 2-dimensional (2D) echocardiography.^9,10^ However, there remains a paucity of data regarding the accuracy of CO measurements by echocardiography in the hands of noncardiologists, in comparison with cardiologists for pediatric patients. Because the guidelines recommend the apical position, currently published studies use the apical/parasternal position for CO and Cardiac index (CI) measurement.^11^ To our knowledge, there are no data comparing transthoracic echocardiography jugular (TTE-J) and transthoracic echocardiography parasternal (TTE-P) SV measurements in pediatric patients. To fill the knowledge gap, we, therefore, decided to conduct a prospective observational study comparing USCOM, TTE-P, and TTE-J measurements by a cardiologist and intensivist, who had defined and limited experience in echocardiography.

## AIMS OF THE STUDY

The specific aims of this study were as follows: (1) to provide a description of the intraoperator variability and interrater reliability of SV measurements using transcutaneous Doppler imaging (USCOM, TTE-J, and TTE-P positions) and (2) to compare the SV measurements by USCOM against SV measurements obtained by TTE in pediatric spontaneously ventilating patients. Both measurements were obtained by cardiologist and noncardiologist with limited training in echocardiography. We hypothesized that the results of the TTE-J measurement would not differ from the TTE-P measurements.

## METHODS

### Study Design and Subjects

We conducted a single-center, prospective observational study at a tertiary pediatric intensive care unit (ICU) at Faculty Hospital in Pilsen, the Czech Republic. The local ethical committee at our institution approved the study.

### Inclusion Criteria

Spontaneously ventilating patients aged from 1 month to 18 years were eligible for the study. The investigator explained the purpose of the study, and written assent was obtained from the subjects or from an authorized representative.

### Exclusion Criteria

Subjects with hemodynamic instability, congenital heart disease with intracardiac shunt physiology, valvular heart disease (including stenosis or insufficiency), tracheostomy tubes (due to inadequate windows obtained with the ultrasound probe), or unable to tolerate the supine position of the study procedures were excluded from the study.

### USCOM 1A

Velocity time integral (VTI) and SV measurements were performed using the USCOM 1A device. This device uses continuous-wave Doppler ultrasound technology with a 3.3-MHz transducer, placed in the suprasternal notch to obtain an optimal flow across the aortic valve to measure left-sided cardiac parameters. The flow from the valve is measured as the VTI-area under the trace. An acceptable cardiac profile must have a well-defined triangular shape with a clear start and cessation of flow (completion of systole), have the highest and sharpest peak achievable, and show the signal filling in of the profile. Once the operator was satisfied with the signal, 2 cardiac profiles were selected from the display for averaging to obtain 1 set of measurements. Each operator obtained 3 sets of USCOM measurements within a period of 3 to 5 minutes. Patient data including height, weight, and sex were entered into the USCOM device at the beginning of the procedure. The outflow tract diameter (OTD) and cross-sectional area of the aortic valve were derived from the height-indexed regression equations.

### Standard Echocardiography

To measure VTI and SV by 2D echocardiography, Doppler flow curves were acquired using a transducer placed in the suprasternal notch (TTE-J) and additionally in the apical long-axis view (TTE-P) to obtain an optimal flow signal (in supine or left lateral position, respectively). Depending on the size of the patient, either a 2- to 4-Hz or 3- to 8-MHz transducer was used. The flow from the aortic valve was measured in both views as the VTI using pulse wave Doppler technique. Once the operator was satisfied with the signal, 2 cardiac profiles were selected from the display for averaging to obtain 1 set of measurements. Each operator obtained 3 sets of echocardiogram Doppler measurements within a period of 3 to 5 minutes.

The aortic valve diameter was measured using 2D echocardiography in the parasternal long-axis view according to the American Society of Echocardiography guidelines.^11^ This was measured through 3 consecutive cardiac cycles and the mean was used to estimate the valve cross-sectional area, using the formula π × (left ventricular outflow tract [LVOT] diameter/2).^2^ The SV of the left ventricle was calculated as a product of aortic valve cross-sectional area multiplied by VTI.

### Physicians

The USCOM monitoring and echocardiography evaluation of the aforementioned parameters was performed by 2 investigators participating in the study. The OP-A was a trained pediatric intensivist widely experienced with USCOM (more than 300 examinations) and with limited previous training in sonography (20 previous supervised examinations, 4 hours of theoretical sessions before the beginning of the study). The OP-B was a certified pediatric cardiologist with limited previous experience with USCOM (35 previous examinations). To ensure blind results, the investigator who was not actively engaged in measuring hemodynamic parameters was absent from the room to prevent visual and auditory bias by the other investigator. Each set of measurements with USCOM was limited to a maximum of 5 minutes. All values (VTI, SV) displayed on the screen were hidden from the observer with a cover to prevent bias. A research assistant recorded VTI, SV, and OTD measurements while the investigator was blinded to these results. After completion of the study, the second operator, blinded to the results of the previous examination, performed the USCOM examination. The echocardiographic examination followed completion of the USCOM study.

### Statistical Analysis

Descriptive summaries of baseline demographic characteristics were generated for all patients included in the study. The applicability of both methods was defined as the percentage of patients with acceptable signal tracings obtained by both raters.

The USCOM and TTE measurements were reported as mean and standard deviation (SD). For reported results comparing paired measurements of OTD and SV that were obtained by USCOM A1 and TTE, Wilcoxon paired test, Bland-Altman analysis (bias and limits of agreement), percent difference analyses, and Spearman correlations were used. The percent difference was calculated as (2 × SD of bias)/mean. All statistical analyses were performed by a licensed statistician using SW SAS (SAS Institute Inc., Cary, NC).

## RESULTS

During the study period, 37 patients were enrolled in the study. Four patients were excluded due to missing data (2 due to unacceptable signals obtained by the ECHO and 2 due to unacceptable signals from the USCOM). Both methods were applicable in 89% of patients. In 33 patients, a total of 39 measurements were obtained with good signal quality. The subjects' characteristics and diagnostic categories are summarized in Table 1.

**TABLE 1:** Subjects' Characteristics

Subjects Characteristics	
Subjects (n)	37
Missing data (No. patients)	4
Analysis (No. patients)	33
Applicability (%)	89
Girls (%)	57
Age (y), mean (SD)	7.55 (4.65)
Weight (kg), mean (SD)	25.67 (14.37)
Height (cm), mean (SD)	1.22 (0.32)
Diagnostic category (%)	
Neurological	11
Hematological	14
Respiratory	14
Infectious	21
Metabolic	16
Traumatic	4
Gastrointestinal	11
Cardiac	3
Surgical	6

Using TTE, OTD measured by OP-A was 1.48 (0.33) with no statistical difference from OTD measured by OP-B (1.49 [0.32]; *P* = 0.2325). The OTD calculated by USCOM was 1.44 (0.33), which statistically differed from OTD measured by TTE in both operators (*P* < 0.01 and *P* < 0.0001).

The SV measured by OP-A using USCOM, TTE-J, and TTE-P were 46.15 (25.48), 39.45 (20.65), and 33.42 (16.69) mL, respectively. The SV measured by OP-B using USCOM, TTE-J, and TTE-P were 43.99 (25.24), 38.91 (19.98), and 37.58 (19.81) mL, respectively. The intraobserver variability of the USCOM measurement was 4.3% in OP-A and 6.3% in OP-B. The intraobserver variability of TTE-J and TTE-P measurements were 4.7% and 4.9% in OP-A and 5.3% and 6.1% in OP-B, respectively.

**FIGURE 1:**
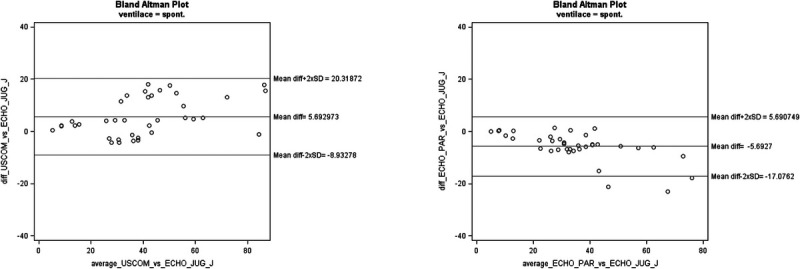
Bland-Altman plots for SV between USCOM versus ECHO-J and ECHO-P versus ECHO J in operator A. For data analysis, 2 outliers were extracted (n = 37).

The bias (and limits of agreement) for SV comparing USCOM to TTE-J in OP-A was 5.69 (−8.93–20.32) and −5.69 (−17.08–5.69) comparing TTE-P to TTE-J (Fig. 1).

**FIGURE 2:**
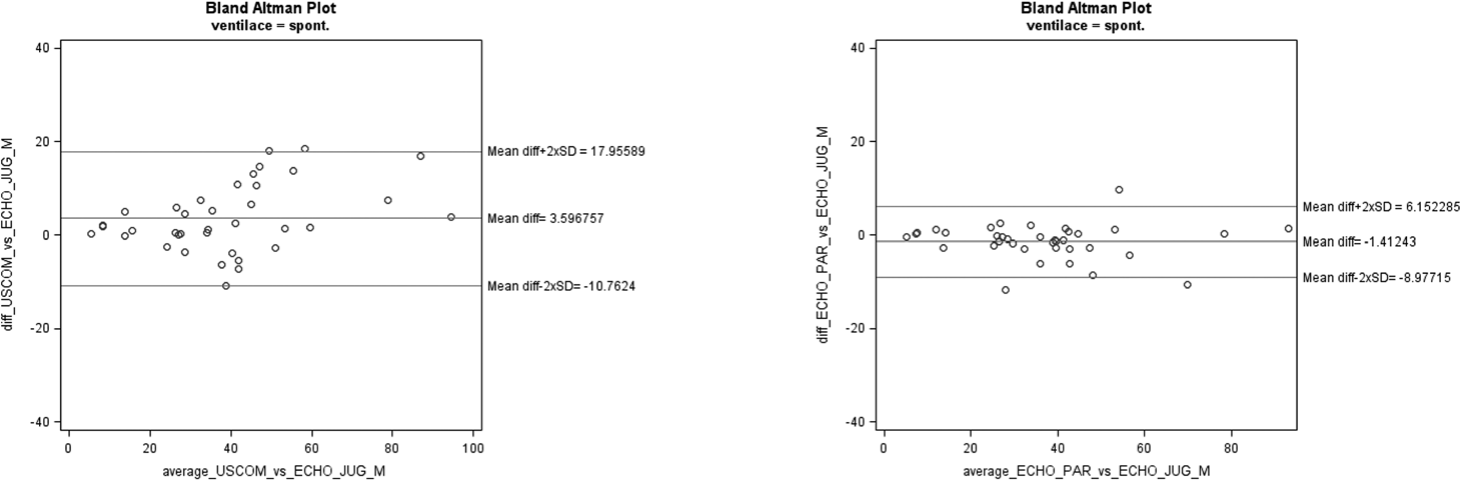
Bland-Altman plots for SV between USCOM versus ECHO-J and ECHO-P vs. ECHO J in operator B. For data analysis 2 outliers were extracted (n = 37).

The bias (and limits of agreement) for SV comparing USCOM to TTE-J in OP-B was 3.60 (−10.76–17.96) and −1.41 (−8.98–6.15) comparing TTE-P to TTE-J (Fig. 2).

The percentage error (PE) in SV measurements with USCOM was 36% relative to TTE-J measurements in OP-A and 37% in OP-B. The PE in SV with TTE-P was 33% relative to TTE-J in OP-A and 21% in OP-B. All data obtained by USCOM and TTE are summarized in Table 2.

**TABLE 2:** USCOM Reliability and Accuracy Analyses (n = 39)

	Operator A	Operator B	*P*
OTD USCOM (cm)	1.44 (0.33)	1.44 (0.33)	NA
OTD ECHO (cm)	1.48 (0.33)	1.49 (0.32)	*P* > 0.05
OTD (USCOM vs ECHO) *P*	<0.01	<0.0001	
Spearman rank correlation	0.88	0.90	
USCOM SV (mL)	46.15 (25.48)	43.99 (25.24)	*P* < 0.01
Spearman rank correlation	0.97		
TTE-J, SV (mL)	39.45 (20.65)	38.91 (19.98)	*P* = 0.4795
Spearman rank correlation	0.92		
TTE-P, SV (mL)	33.42 (16.69)	37.58 (19.81)	*P* < 0.0001
Spearman rank correlation	0.94		
USCOM vs TTE-J	*P* < 0.0001	*P* = 0.0012	
Spearman rank correlation	0.91	0.91	
USCOM vs TTE-P	*P* < 0.0001	*P* < 0.0001	
Spearman rank correlation	0.90	0.91	
TTE-J vs TTE-P	*P* < 0.0001	*P* = 0.0170	
Spearman rank correlation	0.97	0.98	
USCOM, SV			
Intraobserver variability (mL)	2.36 (2.09)	3.37 (3.35)	*P* = 0.1379
Variation coefficient (%)	5.4 (5.6)	7.9 (8.0)	
TTE-J, SV			
Intraobserver variability (mL)	2.19 (1.88)	2.89 (2.76)	*P* = 0.1957
Variation coefficient (%)	5.8 (5.4)	6.5 (4.3)	
TTE-P, SV			
Intraobserver variability (mL)	2.06 (1.82)	2.64 (1.75)	*P* = 0.0414
Variation coefficient (%)	6.2 (4.0)	7.8 (5.8)	
Bland-Altman analysis, bias (limits of agreement)			
USCOM vs TTE-J, SV	5.69 (−8.93–20.32)	3.60 (−10.76–17.96)	
TTE-P vs TTE-J, SV	−5.69 (−17.08–5.69)	−1.41 (−8.98–6.15)	
Percentage difference (%) − SV			
USCOM vs TTE-J, SV	36%	37%	
TTE-P vs TTE-J	33%	21%	

NA indicates not applicable.

## DISCUSSION

Transthoracic echocardiography and USCOM techniques have become widely used methods of CO monitoring in daily pediatric practice (emergency rooms, pediatric ICUs, and general pediatric wards). The novelty of our study is the comparison of USCOM technique with 2 TTE projection measurements (TTE-J and TTE-P, respectively). To our knowledge, this is the first study comparing TTE-J and TTE-P SV measurements. A metaanalysis of studies by Chong^12^ validating the accuracy of CO measurements obtained by USCOM as compared with PAC thermodilution methods showed mixed results; the range of PEs across the studies ranged from 14% to 56%. Several previous studies showed excellent agreement on CO measurements obtained by PAC with those obtained by TTE in different clinical situations.^13,14^

To our knowledge, there are only 2 previous studies comparing CO obtained by USCOM to 2D echocardiography in children.^9,10^ The study by Nguyen et al^9^ included 44 pediatric emergency patients. They showed that the bias and limits of the agreement of USCOM 1A compared with TTE CO was 0.96 (−1.46–3.37) and the percentage difference in CI measurement with USCOM 1A was 41% ± 33% relative to TTE measurements. They concluded that USCOM 1A showed poor correlation and agreement to standard TTE measures of cardiac function.^9^

The study by Wongsirimetheekul et al^10^ included 34 critically ill pediatric ICU patients. They showed that the bias ± precision of USCOM 1A relative to TTE CI was 0.54 ± 1.03 L/min/m^2^, and the percentage of error of the CI measurement with USCOM 1A was 42.3% relative to TTE. They concluded that USCOM was an unreliable tool for absolute value measurements of CO due to the errors of VTI by continuous Doppler.^10^ In accordance with the studies cited previously, our study demonstrates a lower SV measured by echocardiography compared with SV measured by USCOM for both operators. However, the use of Doppler ultrasound techniques to determine CO has several inherent technological limitations. Potential sources of variation exist in the estimation of the aortic outflow tract area and the determination of VTI. Both methods are highly operator-dependent. The aortic outflow tract area is not directly measured with USCOM 1A but calculated from a proprietary anthropometric algorithm based on the patient's height and weight. Using TTE, the aortic outflow tract area is determined by direct measurement of the aortic OTD. The OTD in our study measured with TTE by the cardiologist and intensivist showed a close correlation (r = 0.90) and no statistical difference (*P* = 0.2325). The OTD values measured by both operators using TTE were significantly higher compared with the OTD calculated by USCOM. Nevertheless, the resulting SV values measured by USCOM for both operators were substantially higher compared with the SV values measured using TTE. We conclude that this is a consequence of the use of continuous Doppler measurement used by USCOM, as opposed to the pulse Doppler measurement used in TTE.

The accuracy of the TTE and USCOM 1A technology depends on good flow signal to obtain accurate and reproducible VTI values. In our study, 89% (33/37) of subjects enrolled had acceptable Doppler signal to calculate SV (4 patients were excluded with 2 excluded due to unacceptable signals obtained by ECHO or USCOM, respectively). The applicability in our study does not differ from applicability in previous studies.^9,10^ We also attempted to control for subject position-dependent Doppler signal quality by performing all USCOM 1A and TTE-J measurements in the supine position because positioning has been shown to influence the Doppler signal quality.^15^ Our study included only spontaneously ventilating children who did not require any kind of hemodynamic support in an attempt to ensure a constant CO and to minimize the possible confounding influences.

Because the final SV analysis was calculated as the mean from 3 consecutive measurements, the USCOM and TTE showed intraoperator variability of less than 10% for both operators. This finding is consistent with previous studies and meets the criteria of clinically acceptable variability.^9,10^

Critchley et al^16^ used an error-gram to propose a difference of 30% or less in CI measurements as acceptable, when comparing a new hemodynamic monitoring technique with the reference standard. For the purpose of our study, the TTE-J was selected as a reference method, and thereby, we calculated the PE between USCOM and TTE-J and between TTE-P and TTE-J, respectively. Originally, Critchley et al recommended calculating the PE for CI. Because we used the SV measurements in our study to compare USCOM and TTE techniques, we calculated the PE using the SV measurement results. The PE between USCOM and TTE-J was 39% in OP-A and 46% in OP-B. When the 2 outliers were removed from each calculation, the PE reached 36% and 37%, respectively. The PE between TTE-P and TTE-J was 33% in OP-A and 21% in OP-B. Despite the statistically significant difference in SV measured by TTE-J versus TTE-P in OP-B (*P* = 0.017), the PE between these 2 projections was less than the proposed difference of 30% and thereby is insignificant. This finding leads us to the conclusion that TTE-P and TTE-J are interchangeable if used by an experienced operator.

Previous studies showed that physicians with no previous ultrasonographic experience could be trained to obtain reliable CO estimations on conscious emergency department patients with the USCOM, over the course of less than 50 patient assessments.^8^ Gaspar et al^17^ revealed a significant reduction in the difference in the mean CI measured by the beginners using TTE compared with the mean CI measured by the skilled echocardiographer over the last third of 96 measurements. Comparison of differences between the TTE-J and TTE-P in the first and last 10 TTE SV measurements for the noncardiologist in our study did not show a decreasing degree of difference (TTE-P SV measurement was substantially lower compared with TTE-J SV), which indicates the need for a longer learning period to acquire sufficient skills.

In our study, we demonstrated that a noncardiologist echocardiographer with limited training in cardiac ultrasonography is able to acquire images of sufficient quality and accuracy to allow measurement of SV using TTE-J. Our data clearly demonstrated that the TTE-P SV measurement technique requires longer practice to reach sufficient skill.

## CONCLUSIONS

Our findings demonstrate that USCOM and TTE are not interchangeable methods because SV values by USCOM are higher compared with the SV values obtained by TTE. Both methods have a low level of intraobserver variability. The SV measurements obtained by TTE-P were significantly lower compared with the TTE-J for the operator with limited training in pediatric echocardiography. The TTE-P requires longer practice compared with the TTE-J, and we therefore recommend TTE-J to TTE-P for inexperienced operators in spontaneously ventilating children. Further studies will be required to determine the accuracy of both noninvasive sonographic techniques under changing hemodynamic conditions in both clinical and experimental settings.^18,19^

### Limitations of the Study

We acknowledge several limitations of our study. This was a single-center study. We were not able to make simultaneous assessments of SV by USCOM and TTE due to issues with the techniques and blinding of the investigators. Although we attempted to keep patients at unchanged conditions and steady state and tried to keep the interval between the measurements as brief as possible, it is not possible to rule out physiologic changes of CO over the time of the study. We included only spontaneously ventilating patients; therefore, the results of the study should be limited only to spontaneously ventilating patients who are hemodynamically stable. The conclusions are not applicable to critically ill patients or patients on mechanical ventilation. Compared with other studies, we chose SV and OTD as the main parameters for analysis because these 2 variables directly determine the CO and are directly measured by both monitoring techniques. Critchley et al originally recommended CO and CI for analysis of new and reference CO measurement techniques We did not precisely test the learning curve for any of the techniques.
